# Unilateral Diffuse Alveolar Hemorrhage Due to Selective Directionality of Mitral Regurgitant Jet in a Patient With Severe Aortic Stenosis

**DOI:** 10.7759/cureus.14714

**Published:** 2021-04-27

**Authors:** Santu Saha, Woon H Chong, Biplab K Saha

**Affiliations:** 1 Internal Medicine, Bangladesh Medical College, Dhaka, BGD; 2 Pulmonary and Critical Care Medicine, Albany Medical Center, Albany, USA; 3 Pulmonary and Critical Care Medicine, Ozarks Medical Center, West Plains, USA

**Keywords:** diffuse alveolar hemorrhage, unilateral, cardiac disease, aortic stenosis, mitral regurgitation

## Abstract

Diffuse alveolar hemorrhage (DAH) in cardiac diseases results from pulmonary capillary stress failure due to pulmonary venous hypertension. The most common cardiac causes of DAH are heart failure and mitral valvular disease. Patients typically manifest with hemoptysis, radiologic chest abnormalities, and anemia. The chest infiltrates are generally bilateral, similar to pulmonary edema. Rarely, the chest infiltrates can be unilateral, mimicking an infectious etiology. We present the case of an 88-year-old female with critical aortic stenosis, who presented with shortness of breath, unilateral right lung infiltrates, and mild leukocytosis. The patient was misdiagnosed with pneumonia as pulmonary edema or DAH was expected to be a bilateral finding on chest imaging. The patient deteriorated and DAH was eventually diagnosed by bronchoscopy.

## Introduction

Diffuse alveolar hemorrhage (DAH) is a life-threatening complication of cardiac diseases. Unless promptly identified and treated, it can be rapidly fatal. Regardless of the etiology, the classic presentation of DAH includes hemoptysis, radiologic chest infiltrates, and anemia. The chest infiltrates are typically bilateral, peri-hilar, and mid and lower zone predominant. The pathophysiologic alterations that cause pulmonary edema in cardiac diseases are also responsible for causing DAH. As a result, DAH from pulmonary venous hypertension is classically bilateral. However, a small subset of patients may present with unilateral pulmonary edema (UPE) or DAH, mimicking a focal infectious process or nonresolving pneumonia. Failure to consider this possibility may result in misdiagnosis, ultimately leading to poor patient outcomes. We present a case of unilateral diffuse alveolar hemorrhage (UDAH) due to severe mitral regurgitation in the setting of critical aortic stenosis.

## Case presentation

**Figure 1:**
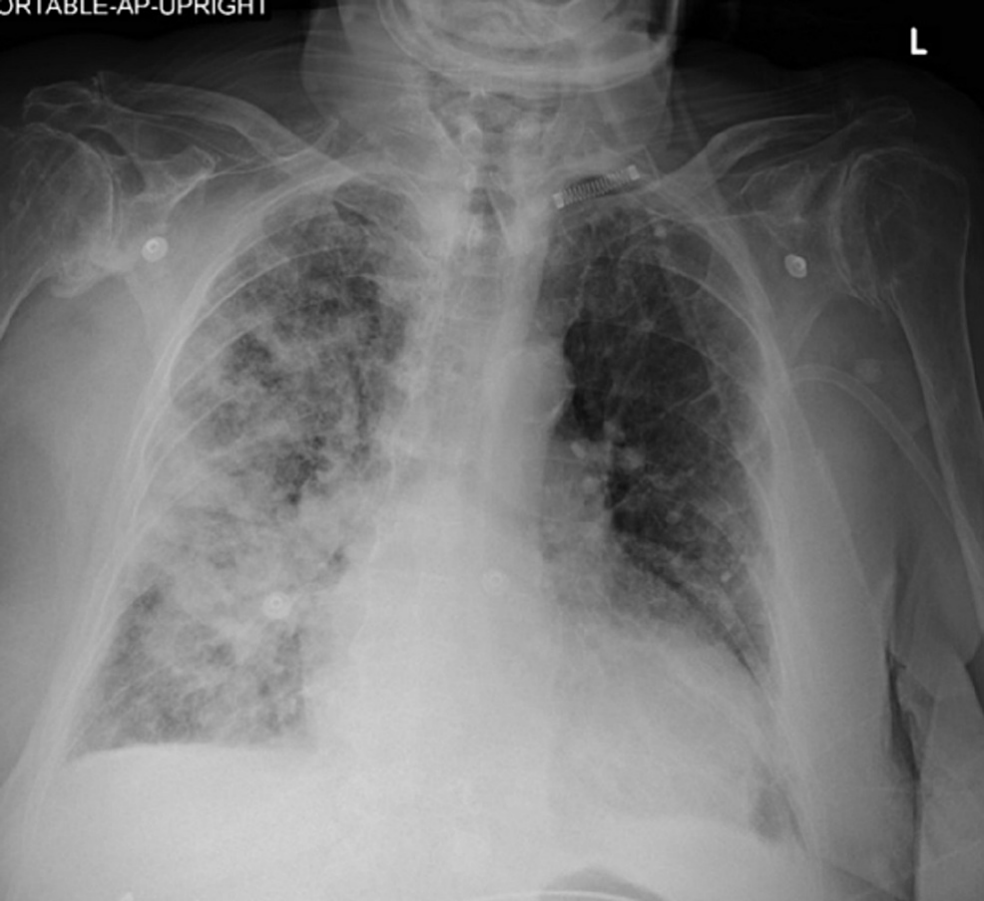
Portable anteroposterior chest X-ray showing alveolar opacity, consolidation, and air bronchogram in the right upper, middle, and lower lung zones. There was significant cardiomegaly. The bilateral costophrenic angles are sharp without any evidence of pleural effusion.

An 88-year-old female presented to the emergency department (ED) with complaints of cough and worsening shortness of breath for 24 hours. Her medical history was significant for severe aortic stenosis with a calculated aortic valve area (AVA) of 0.6 cm^2^ and a mean gradient across the valve of 40 mmHg. In the ED, the patient was tachypneic and in visible respiratory distress. Her oxygen saturation of 86% on room air was corrected with supplemental oxygen. A chest X-ray revealed patchy alveolar opacity with consolidation and air bronchograms in the right upper, middle, and lower lung zones (Figure 1).

**Figure 2:**
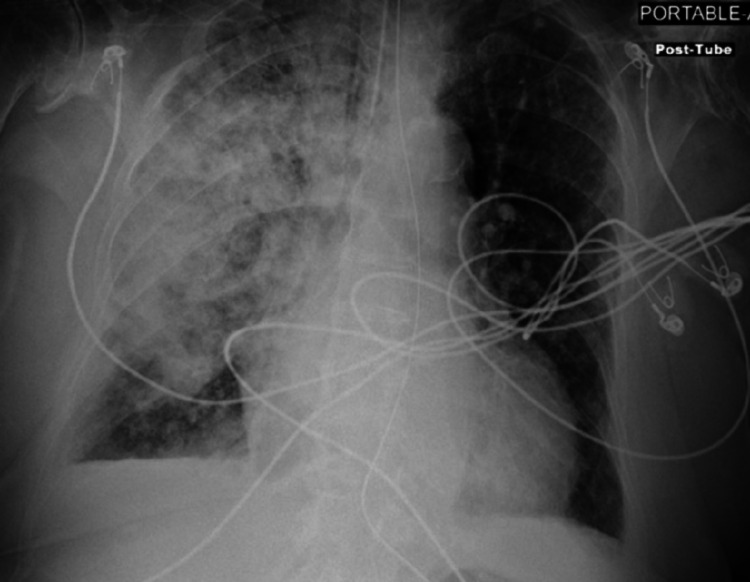
Portable chest radiograph following intubation demonstrated worsening of the right lung infiltrate. The left lung is without any significant abnormality.

**Figure 3:**
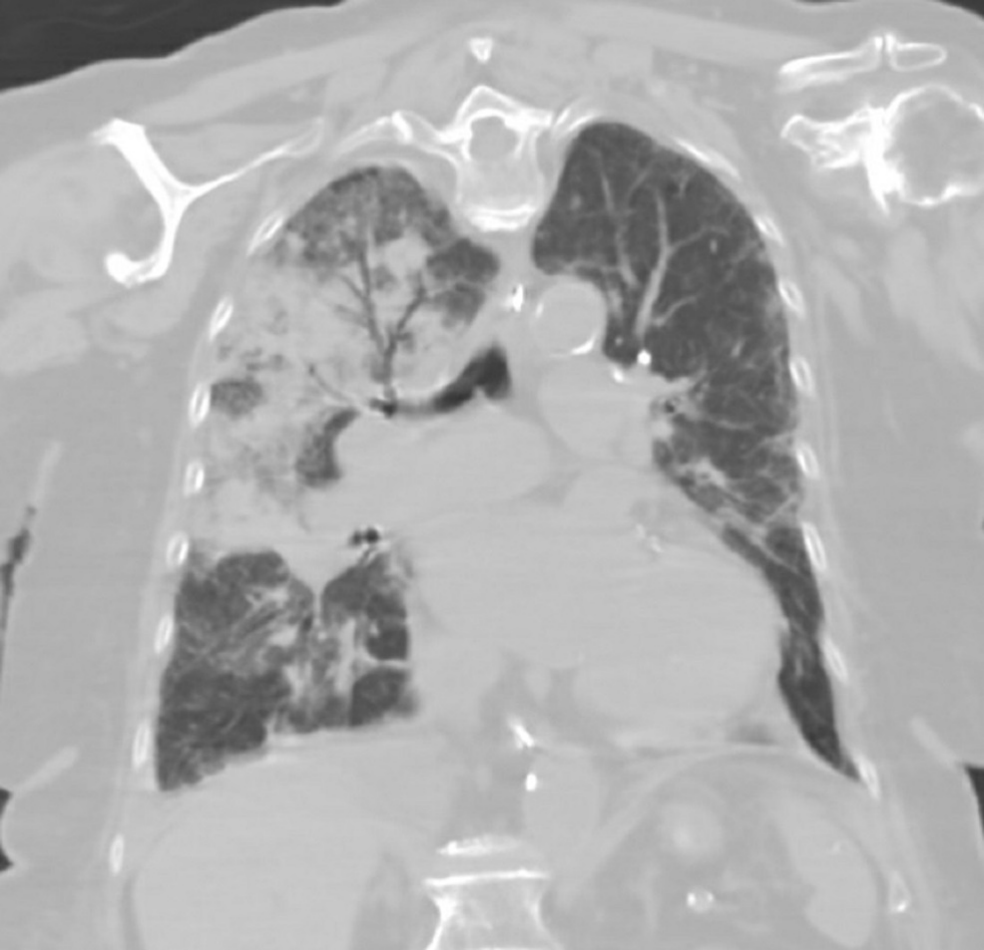
Coronal view of the computed tomography scan of the chest revealed consolidation and air bronchograms involving the right upper lobe.

**Figure 4:**
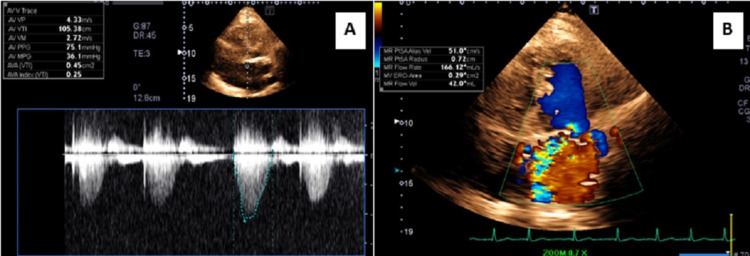
Echocardiogram showing severe aortic stenosis (A). Continuous-wave Doppler in apical five-chamber view showed a peak aortic jet velocity of 4.3 m/sec with the calculated aortic valve area of 0.45 cm2. (B) Color Doppler in apical four-chamber view revealed severe mitral regurgitation. The eccentric regurgitant jet was noted to be selectively directed towards the right pulmonary veins.

**Figure 5:**
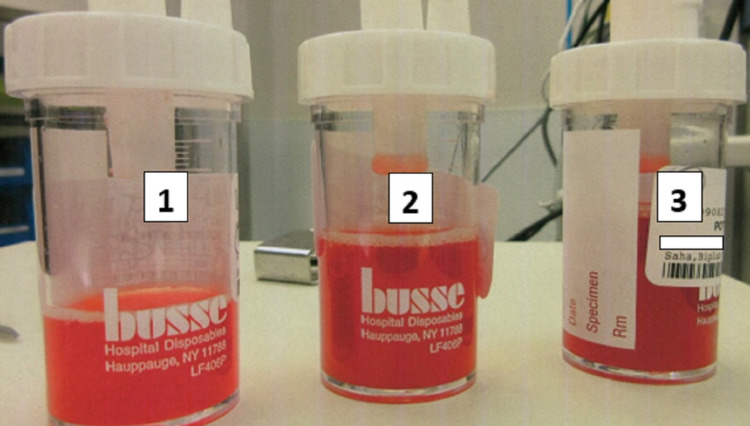
Serial aliquots during bronchoalveolar lavage demonstrated a progressively bloody return. The numbers on the tube correspond to the sample obtained by sequential bronchoalveolar lavage.

Laboratory workup was significant for leukocytosis (18.3 × 103/µL). Her hemoglobin on admission was 14.1 gm/dL. She was diagnosed with community-acquired pneumonia and started on broad-spectrum antibiotics and intravenous fluid. Over the next 36 hours, her respiratory status worsened. The patient was intubated after failing noninvasive positive pressure ventilation for worsening hypoxic respiratory failure (Figure 2). Computed tomography (CT) scan of the chest revealed right upper lobe consolidation with air bronchograms (Figure 3). An echocardiogram showed worsening of aortic stenosis (AVA 0.4 cm^2^) and severe mitral regurgitation (Figure 4). The eccentric regurgitant jet was directed towards the right pulmonary veins. Bedside bronchoscopy revealed blood in the airways (predominantly on the right side). Bronchoalveolar lavage (BAL) demonstrated progressively bloody fluid return from the apical segment of the right upper lobe (RUL) (Figure 5). A comprehensive microbiologic workup was negative. A large quantity of hemosiderin-laden macrophages was identified on BAL cytology, suggestive of DAH. The patient’s hemoglobin level at this point had dropped to 8.5 gm/dL. A right heart catheterization revealed a mean pulmonary artery pressure (mPAP) of 55 mmHg and a pulmonary artery occlusion pressure (PAOP) of 32 mmHg with a large ‘V’ wave. The calculated cardiac index was 2.1 L/min/m^2^ by the thermodilution method. An autoimmune workup including antinuclear antibody (ANA), antineutrophil cytoplasmic antibody (ANCA), and rheumatoid factor (RF) was negative. The patient was aggressively diuresed with pressor support. She was eventually liberated from mechanical ventilation and discharged from the hospital (as per the patient's request) with the plan to undergo prompt outpatient evaluation for transcatheter aortic valve replacement.

## Discussion

DAH due to cardiac diseases is an under-recognized phenomenon. Common cardiac diseases associated with DAH include heart failure, mitral valve disease, and rarely, aortic valve disease. The incidence of DAH due to heart failure varies between 1 and 3.3 cases per year in tertiary academic centers, and cardiac causes are responsible for approximately 30% of hospitalized DAH cases [1]. The elevated left atrial pressure (either in the setting of chronically elevated left ventricular end-diastolic pressure in heart failure or due to acute mitral valve regurgitation) is transmitted to the pulmonary circulation. The DAH occurs due to elevated transmural pulmonary capillary pressure leading to the stress failure of the capillary wall [2]. Additionally, congestion of bronchial circulation secondary to backpressure and impaired forward flow to the left atrium may also result in pulmonary hemorrhage.

The capillary stress failure results from a progressive increase in hydrostatic pressure. An increase in pulmonary capillary hydrostatic pressure initially gives rise to perivascular leakage of plasma ultrafiltrate, causing pulmonary edema. As the pressure continues to rise, serum macromolecules can leak into the interstitial space by "pore stretching" without damaging pulmonary capillary endothelium [1]. Once the transmural capillary pressure reaches a critical point, endothelial damage and DAH ensues. Therefore, cardiogenic pulmonary edema and DAH can be considered two opposite extremes along a spectrum of pulmonary manifestations. A transmural pulmonary capillary pressure of 40 mmHg has been consistently shown to cause endothelial damage in experimental animal studies [3]. Although the actual measurement of transmural pulmonary capillary pressure is not feasible in humans, the value is thought to be between the mPAP and PAOP (possibly closer to mPAP) [4].

Classically, DAH manifests with hemoptysis, radiologic chest infiltrates, and anemia [5,6]. However, the complete triad is present in only a minority of patients. Other symptoms include cough, chest pain, fever, fatigue, and shortness of breath. Radiologically, DAH typically presents with bilateral pulmonary infiltrates in the peri-hilar distribution, predominantly in the mid and lower lung zones [7]. There is often subpleural sparing [8]. A CT scan of the chest is more sensitive than conventional chest radiography and may show additional radiologic abnormalities not seen on a chest X-ray. Ground glass opacity in the same distribution is typically seen in DAH. Moreover, interlobular septal thickening, crazy paving patterns, and sometimes centrilobular nodules may also be present [9]. It is crucial to point out that DAH may also cause airspace consolidation with air bronchograms. Differentiation between pulmonary edema and DAH may be challenging as many radiologic abnormalities can be present in both. However, the presence of consolidation and air bronchograms are more likely to be associated with DAH [10].

Unilateral diffuse alveolar hemorrhage is a unique manifestation of DAH from cardiac causes. The true incidence of UDAH is currently unknown. Although the pathophysiologic mechanism behind the development of UDAH and UPE is similar, the incidence of UDAH is likely much lower. A retrospective study may provide further insight into the rarity of UDAH [11]. In this study spanning eight years, the prevalence of UPE was only 2.1% among all patients admitted with cardiogenic pulmonary edema. Approximately 90% of these patients had right-sided infiltrate [11]. As data regarding the occurrence of UDAH are scarce (mostly in the form of case reports), a diagnosis is often delayed or even missed, as was the case for our patient [12]. The following mechanisms have been proposed as the possible explanation for the preferential occurrence of right-sided DAH: (i) *Directionality of the regurgitant flow*: Severe mitral regurgitation is often present in this patient population. The predominantly RUL DAH is caused by the regurgitant jet directed towards the RUL pulmonary vein, increasing the starling forces in this particular location [13,14]. Sometimes, DAH affects the right middle lobe (RML) as well. This can occur in patients with an accessory RML pulmonary vein that drains to the left atrium in a venous confluence with RUL pulmonary vein. Unilateral infiltrate involving the left upper lobe has also been reported [12]. (ii) *Inadequate lymphatic drainage of the right lung*: the lymphatic drainage of the right and left lungs is accomplished by the right broncho-mediastinal trunk and thoracic duct, respectively. It has been theorized that the larger size of the thoracic duct allows for faster clearance of the interstitial fluid collection from the left lung, leaving the right lung more vulnerable for such occurrence [15]. (iii) *Impediment of the left pulmonary circulation*: the elevated left-sided cardiac filling pressures may potentiate the enlargement of the left-sided structures, which can physically impede the circulation through the left pulmonary artery. The increased blood flow to the right side can cause vascular congestion and increase the propensity for UDAH [16]. (iv) *Variation in pulmonary venous drainage*: impaired venous return from the right lung may also be responsible for UDAH [12].

The diagnosis of DAH is confirmed by demonstrating progressively bloody fluid return with serial aliquots during BAL. Exclusion of infections, inflammatory disorders, drug or toxic exposure, and malignancy is crucial. The cytology shows the predominance of hemosiderin-laden macrophages. Patients with DAH due to a cardiac etiology are treated with supportive therapy and optimization of cardiac filling pressures. This is usually achieved by judicial use of diuretics, vasopressors, and sometimes, with appropriate mechanical interventions [17].

## Conclusions

Unilateral diffuse alveolar hemorrhage is a rare manifestation of cardiac diseases. Although most patients have RUL involvement, a minority of patients may demonstrate left upper lobe involvement as well. The UDAH is likely caused by the selective directionality of the mitral regurgitant flow towards the RUL pulmonary vein. Other mechanisms have also been implicated. The absence of radiologic improvement with diuresis as well as the presence of consolidation with air bronchogram may be more suggestive of DAH than pulmonary edema.

## References

[1] Saha BK, Chong WH (2021). Diffuse alveolar hemorrhage in cardiac diseases. Lung.

[2] West JB, Tsukimoto K, Mathieu-Costello O, Prediletto R (1991). Stress failure in pulmonary capillaries. J Appl Physiol (1985).

[3] West JB, Mathieu-Costello O (1995). Vulnerability of pulmonary capillaries in heart disease. Circulation.

[4] Younes M, Bshouty Z, Ali J (1987). Longitudinal distribution of pulmonary vascular resistance with very high pulmonary blood flow. J Appl Physiol (1985).

[5] Saha BK (2021). Idiopathic pulmonary hemosiderosis: a state of the art review. Respir Med.

[6] Saha BK, Milman NT (2020). Idiopathic pulmonary hemosiderosis: a review of the treatments used during the past 30 years and future directions [PREPRINT]. Clin Rheumatol.

[7] Primack SL, Miller RR, Müller NL (1995). Diffuse pulmonary hemorrhage: clinical, pathologic, and imaging features. AJR Am J Roentgenol.

[8] Chong WH, Saha BK, Austin A, Chopra A (2021). The significance of subpleural sparing in CT chest: a state-of-the-art review. Am J Med Sci.

[9] Cheah FK, Sheppard MN, Hansell DM (1993). Computed tomography of diffuse pulmonary haemorrhage with pathological correlation. Clin Radiol.

[10] Woolley K, Stark P (1999). Pulmonary parenchymal manifestations of mitral valve disease. Radiographics.

[11] Attias D, Mansencal N, Auvert B (2010). Prevalence, characteristics, and outcomes of patients presenting with cardiogenic unilateral pulmonary edema. Circulation.

[12] Tamai K, Tomii K, Nakagawa A, Otsuka K, Nagata K (2015). Diffuse alveolar hemorrhage with predominantly right-sided infiltration resulting from cardiac comorbidities. Intern Med.

[13] Alarcón JJ, Guembe P, de Miguel E, Gordillo I, Abellás A (1995). Localized right upper lobe edema. Chest.

[14] Gurney JW, Goodman LR (1989). Pulmonary edema localized in the right upper lobe accompanying mitral regurgitation. Radiology.

[15] Akiyama K, Suetsugu F, Hidai T, Shimamoto K, Takahashi S (1994). Left-sided unilateral pulmonary edema in postinfarction ventricular septal rupture. Chest.

[16] Schnyder PA, Sarraj AM, Duvoisin BE, Kapenberger L, Landry MJ (1993). Pulmonary edema associated with mitral regurgitation: prevalence of predominant involvement of the right upper lobe. AJR Am J Roentgenol.

[17] Handagala R, Ralapanawa U, Jayalath T (2018). Unilateral pulmonary edema: a case report and review of the literature. J Med Case Rep.

